# Fluorescent Risedronate Analogues Reveal Bisphosphonate Uptake by Bone Marrow Monocytes and Localization Around Osteocytes In Vivo

**DOI:** 10.1359/jbmr.091009

**Published:** 2009-10-12

**Authors:** Anke J Roelofs, Fraser P Coxon, Frank H Ebetino, Mark W Lundy, Zachary J Henneman, George H Nancollas, Shuting Sun, Katarzyna M Blazewska, Joy Lynn F Bala, Boris A Kashemirov, Aysha B Khalid, Charles E McKenna, Michael J Rogers

**Affiliations:** 1 Bone and Musculoskeletal Research Programme, Institute of Medical Sciences, University of AberdeenUK; 2 Warner Chilcott PharmaceuticalsMason, OH, USA; 3 MDS Pharma ServicesBothell, WA, USA; 4 Department of Chemistry, University at Buffalo, The State University of New YorkBuffalo, NY, USA; 5 Department of Chemistry, University of Southern CaliforniaLos Angeles, CA, USA

**Keywords:** bisphosphonates, fluorescent conjugates, cellular uptake, osteocytes, monocytes

## Abstract

Bisphosphonates are effective antiresorptive agents owing to their bone-targeting property and ability to inhibit osteoclasts. It remains unclear, however, whether any non-osteoclast cells are directly affected by these drugs in vivo. Two fluorescent risedronate analogues, carboxyfluorescein-labeled risedronate (FAM-RIS) and Alexa Fluor 647–labeled risedronate (AF647-RIS), were used to address this question. Twenty-four hours after injection into 3-month-old mice, fluorescent risedronate analogues were bound to bone surfaces. More detailed analysis revealed labeling of vascular channel walls within cortical bone. Furthermore, fluorescent risedronate analogues were present in osteocytic lacunae in close proximity to vascular channels and localized to the lacunae of newly embedded osteocytes close to the bone surface. Following injection into newborn rabbits, intracellular uptake of fluorescently labeled risedronate was detected in osteoclasts, and the active analogue FAM-RIS caused accumulation of unprenylated Rap1A in these cells. In addition, CD14^high^ bone marrow monocytes showed relatively high levels of uptake of fluorescently labeled risedronate, which correlated with selective accumulation of unprenylated Rap1A in CD14^+^ cells, as well as osteoclasts, following treatment with risedronate in vivo. Similar results were obtained when either rabbit or human bone marrow cells were treated with fluorescent risedronate analogues in vitro. These findings suggest that the capacity of different cell types to endocytose bisphosphonate is a major determinant for the degree of cellular drug uptake in vitro as well as in vivo. In conclusion, this study shows that in addition to bone-resorbing osteoclasts, bisphosphonates may exert direct effects on bone marrow monocytes in vivo. © 2010 American Society for Bone and Mineral Research

## Introduction

Bisphosphonates (BPs) are antiresorptive drugs that are used widely in the treatment of bone diseases involving excessive osteoclastic resorption.(1) Owing to their two phosphonate groups, BPs bind to bone mineral and are internalized by osteoclasts during the resorption process.(2) Nitrogen-containing BPs (N-BPs), such as risedronate (RIS), disrupt osteoclast function by inhibiting the enzyme farnesyl pyrophosphate (FPP) synthase.(3,4) This causes a depletion of intracellular isoprenoid lipids required for the prenylation of proteins, such as small GTPases, resulting in the accumulation of unprenylated proteins with defective function within the cell.(5,6)

The exact localization of BP within bone, as well as the ability of cells other than osteoclasts to take up BP in vivo, remains unclear. Previous studies have used radiolabeled BPs to study the localization and cellular uptake of BP in animal models.(7–9) Intracellular uptake of [^3^H]alendronate ([^3^H]ALN) was detected in osteoclasts but not any other cells in histologic sections of femora from newborn rats following administration of 0.4 mg/kg [^3^H]ALN.(7) More recently, we and others have developed fluorescent BP analogues to study the localization and cellular uptake of these drugs in more detail. A comparison between Alexa Fluor 488-ALN and [^14^C]zoledronate revealed that these compounds have an identical mechanism of cellular uptake that largely depends on fluid-phase endocytosis,(10) demonstrating that fluorescent BP analogues are useful tools with which to study cellular uptake of BP. More recently, we have used fluorescein-ALN to evaluate intracellular uptake of BP in vitro under a variety of cell culture conditions, demonstrating that fluorescein-ALN binds avidly to bone mineral in vitro and that only osteoclasts efficiently internalize BP from the bone surface during resorption.(2)

Despite these observations, it has become clear in recent years that other cell types may be affected by BPs in vivo. Numerous studies have suggested that effects on tumor cells or endothelial cells may explain the antitumor activity of these drugs in animal models of cancer.(11) In addition, in vitro studies have suggested that effects on circulating monocytes and subsequent activation of peripheral blood Vγ9Vδ2 T cells are responsible for the development of an acute-phase response commonly observed in patients receiving intravenous N-BP administration for the first time.(12–16) It is still unclear, however, which cell types are able to internalize BP and thus are likely to be directly affected by these drugs *in vivo*.

Zaheer and colleagues recently described a novel purified fluorescent BP analogue, IRDye78-labeled pamidronate,(17) and the near-infrared analogues of pamidronate Osteosense680 and Osteosense750 have been evaluated recently for their potential as optical markers of bone metabolism.(18) However, no detailed histologic analysis of localization or assessment of intracellular uptake of these fluorescent BP analogues in vivo has been reported.

We have recently developed a novel method for the synthesis of stable and highly purified fluorescent RIS analogues, including a carboxyfluorescein conjugate (FAM-RIS) that retains the ability of the parent BP to inhibit protein prenylation in cells.(19) In this study, we used both FAM-RIS and Alexa Fluor 647–labeled RIS (AF647-RIS) to investigate in more detail the localization and cellular uptake of BP in vivo.

## Materials and Methods

### Synthesis and purification of fluorescent RIS analogues

RIS was provided by Procter & Gamble Pharmaceuticals (Mason, OH, USA). Fluoresceins and AF647 were purchased from Invitrogen (Paisley, UK). FAM-RIS was synthesized by stable conjugation of 5(6)-carboxyfluorescein [5(6)-FAM] or its pure isomers 5-FAM or 6-FAM via its succinimidyl ester to the pyridine nitrogen of RIS via a novel linker strategy and highly purified, as described previously.(19) AF647-RIS was synthesized similarly. Drugs were dissolved in PBS.

### Constant composition kinetic studies of crystal growth

Constant composition (CC) hydroxyapatite (HAP) growth experiments were performed as described previously(20) in the presence of 1.0 µM BP. Carbonated hydroxyapatite (CAP), prepared by direct precipitation from solution,(21) was used as seed material; CAP crystals contained 7.96% carbonate by mass, as determined by carbon coulometry.

### Treatment of animals

All animal studies were approved by the Home Office (UK) and conducted in accordance with the Animals (Scientific Procedures) Act of 1986 and the Home Office Code of Practice. To study the localization of BP in vivo, 3 month old C57Bl/6 or MF1 mice received single subcutaneous injections with 0.2 to 1 mg/kg FAM-RIS (molecular weight 714 g/mol) or 0.9 mg/kg AF647-RIS (molecular weight 1198 g/mol). To study cellular uptake in vivo, newborn (3 day old) New Zealand White rabbits received a single subcutaneous injection with 0.5 or 3 mg/kg FAM-RIS, 0.9 mg/kg AF647-RIS, or 1.2 or 5 mg/kg RIS. As controls, animals received a subcutaneous injection of vehicle only, or 0.5 mg/kg unconjugated fluorescein (Sigma Chemical Co., St. Louis, MO, USA). Twenty-four hours or 7 days later, animals were sacrificed and tissues collected.

### Detection of AF647-RIS in whole tissues

Bones and soft tissue organs (kidneys, liver, spleen) of 3 month old female MF1 mice subcutaneously injected with AF647-RIS (0.9 mg/kg) or vehicle were excised either 1 or 7 days after injection. Following fixation, bones and organs were scanned on a LI-COR Odyssey Infrared Imager (LI-COR Biosciences, Cambridge, UK) using the 680 nm laser to detect AF647-RIS.

### Histologic analysis

Mouse tibiae or rabbit ulnae were fixed in 4% formaldehyde and embedded in methyl methacrylate and cut in half either longitudinally or cross-sectionally through the midshaft. Polished block surfaces either were analyzed directly or sections were cut (2 µm section thickness) using a diamond knife. Sections were counterstained with the nuclear dye TO-PRO-3 and/or wheatgerm-agglutinin-Alexa Fluor 594 (Invitrogen) and mounted using Vectashield (Vector Labs, Peterborough, UK) or ProLong Gold (Invitrogen). Blocks and sections were analyzed using a Zeiss LSM510 META system and AIM software or, for acquisition of tile scans to generate high-magnification overview images, on a Zeiss LSM710 META system with ZEN software (Carl Zeiss, Ltd., Welwyn Garden City, UK). For quantification of AF647-RIS labeling of osteocyte lacunar walls, the cortices in cross sections of tibiae from four different mice were analyzed. At least three images of different regions throughout the cortical bone were obtained from each block using a 40× oil objective with identical scaling, scan speed, and detector gain (employing a setting that ensured that there was no saturation of the fluorescence in any of the osteocyte lacunae). Mean fluorescence intensity of each lacunar wall was determined by drawing around the entirety of the lacunae using the line-profile tool, and the distance to the nearest labeled bone or vascular channel surface was measured using AIM software. A total of 632 osteocyte lacunae were analyzed (at least 64 per mouse). Mean fluorescence intensities of osteocyte lacunar walls of two control mice were used to correct for autofluorescence, whereas the maximum background fluorescence measured was used to set a threshold to determine the percentage of osteocyte lacunae that were labeled with AF647-RIS.

### Isolation of rabbit and human bone marrow cells

Bone marrow cells were isolated from the long bones of vehicle-, FAM-RIS-, AF647-RIS-, or RIS-treated rabbits by scraping out the marrow and mincing the bones, as described previously.(22) The use of human bone marrow was approved by the North East Scotland Research Ethics Committee. Informed consent was obtained for the collection of bone marrow from patients undergoing total hip replacement surgery in accordance with the Declaration of Helsinki. Bone marrow was obtained by aspiration during surgery. Red blood cells were lysed using PharmLyse (BD Biosciences, Oxford, UK).

### Isolation of rabbit osteoclasts and monocytes from bone marrow

Osteoclasts were isolated from rabbit bone marrow by using anti-vitronectin receptor (anti-VNR) magnetic beads, as described previously.(23) In some experiments, the VNR-negative fraction (osteoclast-depleted bone marrow cells) was further separated by CD14 MACS magnetic beads (Miltenyi Biotec, Surrey, UK) according to the manufacturer's instructions with minor modifications. To enhance the purity, 4 µL anti-CD14 magnetic beads were used instead of the recommended 20 µL per 10^7^ cells. In addition, the positive (CD14^+^) fraction was passed through a second MACS MS column to further enhance purity. The purity was approximately 90%, as determined by flow cytometric analysis of CD14 staining.

### In vitro treatment of bone marrow cells with fluorescent RIS analogues

For in vitro treatment with fluorescent BPs, rabbit or human bone marrow cells were plated out at 5 × 10^5^ cells/mL in 24- or 48-well plates in medium (RPMI1640 supplemented with 10% FCS, 2 mM glutamine, 100 U/mL penicillin, and 100 mg/mL streptomycin) and left to settle for 2 hours. Cells then were treated with 0 to 1 µM FAM-RIS or AF647-RIS or with 10 µg/mL FITC-dextran for 24 hours.

### Flow cytometry and confocal microscopy

For detecting intracellular uptake of fluorescent RIS analogues by osteoclasts in vivo, osteoclasts obtained by anti-VNR magnetic bead isolation were resuspended in medium (α-MEM with 10% FCS, 2 mM glutamine, 100 U/mL penicillin, and 100 mg/mL streptomycin). Cells were seeded onto glass coverslips in 48-well plates and left to adhere for 4 hours. Cells were washed once in PBS, counterstained with 1 µg/mL TO-PRO-3 or sytox green, and analyzed for intracellular FAM-RIS or AF647-RIS uptake on a Zeiss LSM510 META system. For detecting intracellular uptake of fluorescent RIS analogues or FITC-dextran by other cells, rabbit or human bone marrow cells were stained with either FITC- or APC-conjugated anti-CD14 antibodies (Miltenyi Biotech, Surrey, UK) and analyzed on a FACSCalibur flow cytometer using CellQuestPro software (BD Biosciences). Vehicle-treated cells served as controls for background fluorescence. The mean fluorescence intensity was determined by calculating geometric mean fluorescence in the FL1 (FAM-RIS) or FL4 (AF647-RIS) channels using CellQuestPro software. In some experiments, CD14^high^ AF647-RIS^+^ cells were sorted on a FACSDiVa (BD Biosciences) to facilitate subsequent analysis by microscopy. Cells then were cytospun onto slides and counterstained with 0.5 µg/mL sytox orange prior to analysis on a Zeiss LSM510 META system using AIM software (Carl Zeiss, Ltd.) for image acquisition and analysis.

### Detection of unprenylated Rap1A by Western blotting

Cells isolated by anti-VNR and/or anti-CD14 magnetic bead separation were lysed in radioimmunoprecipitation assay buffer [PBS containing 1% (v/v) nonidet P40, 0.1% (w/v) SDS, 0.5% (w/v) sodium deoxycholate, and 1:100 Sigma protease inhibitor cocktail]. Equal protein amounts then were electrophoresed under reducing conditions on 12% polyacrylamide-SDS gels (Criterion, Bio-Rad, Hemel Hempstead, UK) and transferred onto polyvinyl difluoride membranes by semidry transfer. Blots were incubated with a polyclonal goat anti-Rap1A antibody (Santa Cruz Biotechnology, Santa Cruz, CA, USA) that binds with high affinity to the unprenylated form of this small GTPase(24) and a rabbit monoclonal anti-Rap1 antibody (Cell Signaling Technology, Danvers, MA, USA). Blots then were incubated with Alexa Fluor 680- and IRDye800-conjugated secondary antibodies and analyzed on a LI-COR Odyssey Infrared Imager (LI-COR Biosciences).

### Statistical analysis

Statistical analysis was carried out using two-way analysis of variance and the Holm Sidak posttest using SigmaPlot software.

## Results

### The effects of RIS and fluorescent RIS analogues on HAP crystal growth in vitro

**Fig. 1:**
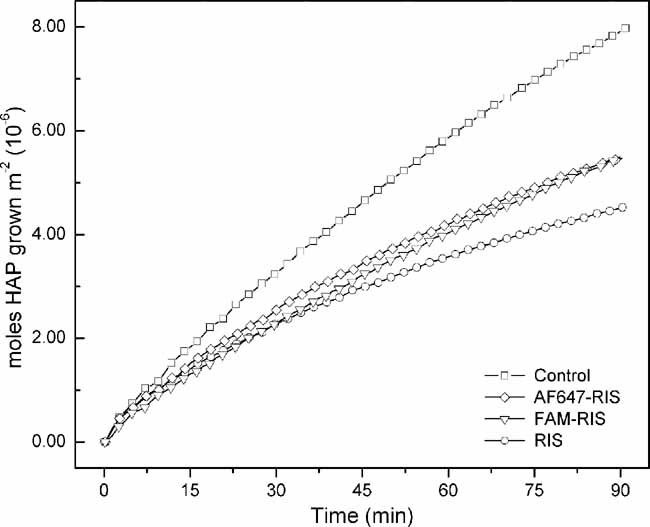
Growth of HAP on CAP in the presence of fluorescent RIS analogues in vitro. Constant composition (CC) crystal growth experiments were conducted in the presence of 1.0 µM BP in order to assess inhibitory potency of fluorescent RIS analogues (AF647-RIS, FAM-RIS) compared with the parent molecule (RIS). Experimental conditions were δHAP (relative supersaturation) = 9.00, pH 7.40 at 37.0°C, and IS (ionic strength) = 0.15 M.

In order to mimic bone mineral, CAP seed was used as a model of mature bone, which has been shown to contain 4% to 7% carbonate by mass.(21,25) Inhibition data for HAP growth on CAP in the presence of fluorescent RIS analogues were compared with unlabeled RIS in an in vitro HAP crystal growth assay, as described previously.(20) The HAP growth rates, corrected for specific surface area (SSA = 60.870 m^2^/g) of the seed material, were 5.57 × 10^−8^ and 5.79 × 10^−8^ mol/m^2^ per minute for AF647-RIS and FAM-RIS, respectively, which is a 35.4% and 32.8% inhibition compared with the growth rate of 8.62 × 10^−8^ mol/m^2^ per minute of the control (Fig. 1). The inhibition achieved by unlabeled RIS was 48.6% (growth rate 4.43 × 10^−8^ mol/m^2^ per minute; see Fig. 1).

### Detection of AF647-RIS in bones and soft tissues

To assess the tissue distribution of fluorescently labeled RIS, the tibia/femur, kidneys, liver, and spleen were dissected from 3 month old MF1 mice that had been injected with AF647-RIS (0.9 mg/kg) either 1 or 7 days previously, and AF647-RIS was detected using a LI-COR Odyssey Infrared Imager with a 680 nm laser. One day after injection, AF647-RIS was clearly detectable in skeletal tissue, with the tibia/femur showing an approximately 100-fold higher average fluorescence intensity than control (*p* < .001; 2*A, B*). AF647-RIS also was present in the kidneys, although at much lower levels (average fluorescence intensity approximately fourfold higher than control, *p* < .05), whereas no significant accumulation in liver or spleen was detected (see 2*A, B*). At 7 days after injection, AF647-RIS still was clearly present in tibia/femur (*p* <.001 compared with control), whereas AF647-RIS was not detectable in kidneys, liver, or spleen (see 2*C, D*). AF647-RIS was not detectable in heart, lung, or eyes at either time point (data not shown).

**Fig. 2:**
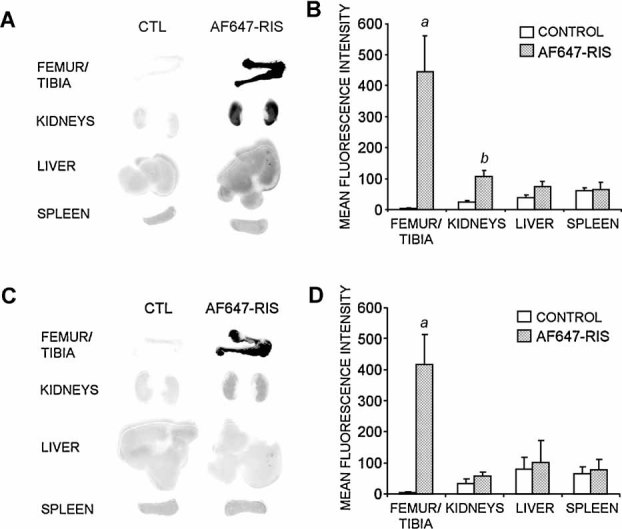
Detection of AF647-RIS in whole tissues. Three month old mice were subcutaneously injected with AF647-RIS (0.9 mg/kg), and the tibia, femur, kidneys, liver, and spleen were dissected either 1 (*A*, *B*) or 7 days after injection (*C*, *D*). Following fixation overnight, bones and organs were scanned on a LI-COR Odyssey Infrared Imager (LI-COR Biosciences) using a 680 nm laser to detect the presence of AF647-RIS. (*A*, *C*) Near-infrared fluorescence images of tissues from control and AF647-RIS-treated mice. (*B*, *D*) Average fluorescence intensity was quantified using Odyssey Version 2.1 software (LI-COR Biosciences) and is expressed as mean ± SD (1 day: *n* = 4; 7 days: *n* = 3). *a*: *p* < .001 compared with control; *b*: *p* < .05 compared with control.

### Binding of fluorescent RIS analogues to bone surfaces in vivo

**Fig. 3:**
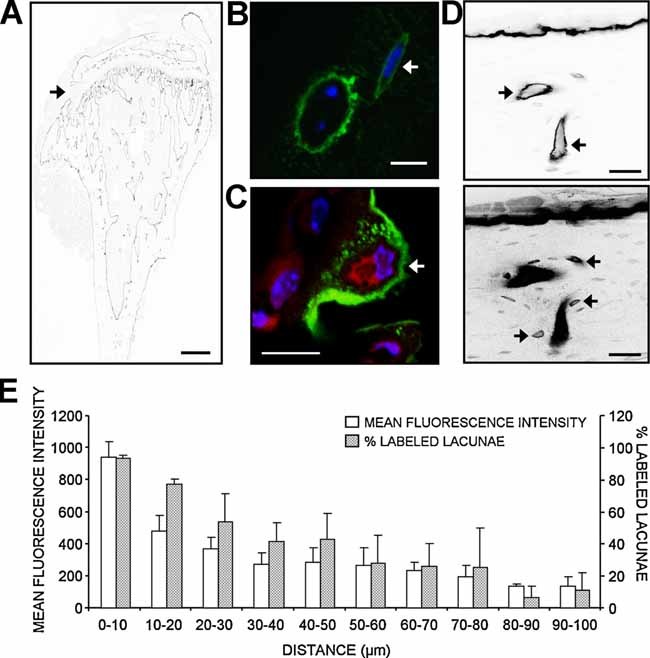
Histologic analysis of FAM-RIS and AF647-RIS binding to bone surfaces in vivo. Three month old mice were injected with 0.5 mg/kg (*A*) or 1 mg/kg (*B*, *C*) FAM-RIS or 0.9 mg/kg AF647-RIS (*D*, *E*) and sacrificed 24 hours later. Tibiae were fixed in formaldehyde, embedded in methyl methacrylate, and sectioned longitudinally (*A–D*) or cross-sectionally through the midshaft (*E*). (*A*) Series of *xy* confocal microscopy images of a 2 µm section were acquired on an LSM710 META system using a 20× objective. Scans were tiled using ZEN software to generate a high-resolution overview image of FAM-RIS (*black*) labeling of bone surfaces. Bar = 500 µm. (*B*, *C*) 2 µm sections were counterstained with the nuclear dye TO-PRO-3 and wheatgerm-agglutinin-Alexa Fluor 594 (*C*) prior to analysis by confocal microscopy. Green: FAM-RIS; blue: TO-PRO-3; red: wheatgerm-agglutinin-Alexa Fluor 594. Arrows indicate the presence of FAM-RIS around osteocytes. Bar = 10 µm. (*D*) *xy* image, acquired directly from the MMA-embedded tibial block just below the block surface, showing AF647-RIS labeling (*black*) within cortical bone near the periosteal surface. *Top image:* Detector gain optimized for detection of AF647-RIS around vascular channels (*arrows*). *Bottom image:* Detector gain optimized for detection of AF647-RIS around osteocytic lacunae surrounding the vascular channels (*arrows*). Bar = 50 µm. (*E*) Quantification of AF647-RIS labeling of osteocyte lacunar walls. The mean fluorescence intensity (corrected for tissue autofluorescence) and the percentage of positively labeled osteocytic lacunae are expressed as a function of the distance to the nearest labeled bone or vascular channel surface (data binned into 10 µm increments). Data shown are the mean ± SEM of four mice (0 to 70 µm) or three mice (70 to 100 µm).

To examine in vivo localization of fluorescent RIS analogues within bone in more detail, mouse tibiae were fixed in formaldehyde and embedded in methyl methacrylate for histologic analysis. Figure 3*A* shows an overview of FAM-RIS labeling in a tibial cross section obtained by tile scanning on a confocal microscope using a 20× objective. At the doses used, virtually all bone surfaces adjacent to marrow space (trabecular and cortical) showed FAM-RIS labeling. Following decalcification of histologic sections, FAM-RIS labeling was no longer detected (data not shown). Unconjugated fluorescein was not detectable in histologic bone sections 24 hours after a single injection of 0.5 mg/kg (data not shown). FAM-RIS labeling was observed only along mineralized bone surfaces and was absent in hypertrophic cartilage of the growth plate (indicated by arrow in 3*A*). Similar findings were obtained with AF647-RIS.

### Localization of fluorescent RIS analogues to vascular channels and osteocytic lacunae

Detailed histologic analysis of mouse and rabbit long bones 24 hours after a single injection with fluorescent RIS analogues revealed localization of FAM-RIS or AF647-RIS around vascular channels and within osteocytic lacunae (see Fig. 3*B–D*). Fluorescently labeled RIS also was observed frequently in canaliculi, including those connecting the vascular channel to the labeled osteocytic lacuna (see 3*B*). In addition, newly forming osteocytic lacunae close to the bone surface showed fluorescent RIS labeling (see 3*C*). Strikingly, osteocytic lacunae in close proximity to vascular channels within the cortical bone showed fluorescent RIS labeling, whereas osteocytic lacunae further away from these channels showed little or no labeling (see 3*D*). Quantification of AF647-RIS labeling confirmed this, showing a clear inverse relationship between the distance to the nearest bone or vascular channel surface and the mean fluorescence intensity of the lacunar wall (corrected for background fluorescence). In addition, the percentage of positively labeled osteocytic lacunae decreased with increasing distance (see 3*E*). There was no evidence of internalization of FAM-RIS or AF647-RIS by osteocytes resident within labeled lacunae.

### Internalization of fluorescent RIS analogues by osteoclasts in vivo

Our initial in vitro studies showed uptake of FAM-RIS from dentine by resorbing osteoclasts.(19) These studies demonstrated accumulation of FAM-RIS within intracellular vesicles in accordance with previous studies demonstrating endocytic uptake of fluorescently labeled ALN by both macrophages and resorbing osteoclasts.(2,10) In this study, we investigated whether intracellular uptake by osteoclasts was detectable in vivo following a single injection with fluorescently labeled RIS. Newborn rabbits were used for these experiments because we demonstrated previously an accumulation of unprenylated Rap1A (indicative of BP uptake) in osteoclasts in vivo following a single injection of neonatal rabbits with RIS.(23) Using this model, FAM-RIS uptake was detected in histologic sections in a small number of large, multinucleated cells close to the bone surface (4*A*). To further confirm uptake by osteoclasts in vivo, osteoclasts were isolated from vehicle-, FAM-RIS-, and AF647-RIS-treated rabbits by VNR magnetic bead separation and analyzed by confocal microscopy. This clearly demonstrated intracellular uptake of FAM-RIS and AF647-RIS into intracellular vesicles by multinucleated VNR-positive osteoclasts (see 4*B*).

**Fig. 4:**
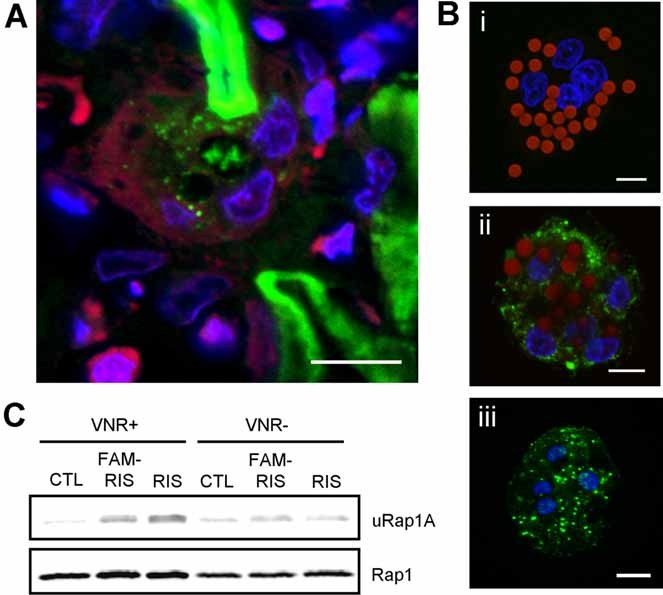
FAM-RIS and AF647-RIS uptake by rabbit osteoclasts in vivo. Newborn rabbits were injected with 0.5 mg/kg FAM-RIS or 0.9 mg/kg AF647-RIS (*A*, *B*) or with 3 mg/kg FAM-RIS or the molar equivalent dose of 1.2 mg/kg RIS (*C*) or vehicle and sacrificed 24 hours later. (*A*) Confocal microscopic image of a longitudinal section through an ulna from a FAM-RIS-treated (*green*) rabbit counterstained with the nuclear dye TO-PRO-3 (*blue*). Tissue autofluorescence shown in red channel. Bar = 10 µm. (*B*, *C*) Bone marrow cells were isolated from the long bones by scraping out the marrow, and osteoclasts were isolated using anti-VNR magnetic beads. (*B*) Isolated osteoclasts were left to adhere on glass coverslips for 4 hours, fixed in formaldehyde, and counterstained with TO-PRO-3 (FAM-RIS and vehicle) or sytox green (AF647-RIS) prior to analysis of intracellular drug uptake using confocal microscopy. *i:* Osteoclast from vehicle-treated rabbit. *ii*: Osteoclast from FAM-RIS-treated rabbit. *Green:* FAM-RIS; *blue:* TO-PRO-3; *red:* Magnetic bead autofluorescence. *iii:* Osteoclast from AF647-RIS-treated rabbit. *Green:* AF647-RIS; *blue:* sytox green. Bar = 10 µm. (*C*) Isolated VNR-positive and VNR-negative cells were lysed and analyzed for the presence of unprenylated Rap1A by Western blotting using an antibody that binds with high affinity to the unprenylated form of the protein (uRap1A) and an antibody that detects total Rap1.

### Inhibition of Rap1A prenylation by FAM-RIS in osteoclasts in vivo

Since FAM-RIS inhibits the prenylation of Rap1A in J774.2 macrophage-like cells in vitro with similar potency to RIS,(19) we investigated the ability of FAM-RIS to inhibit Rap1A prenylation in osteoclasts in vivo as a surrogate marker for cellular uptake. Indeed, the unprenylated form of Rap1A was clearly detectable in the VNR-positive (osteoclast) fraction following treatment with FAM-RIS but was barely detectable in the VNR-negative fraction, similar to unlabeled RIS (see 4*C*). AF647-RIS did not inhibit Rap1A prenylation in J774.2 cells in vitro (data not shown) and therefore was not tested for activity in vivo.

### Uptake of fluorescent RIS analogues by bone marrow cells in vitro

To investigate the possibility that non-osteoclast bone marrow cells may internalize BP in vivo, we first examined the ability of bone marrow cells to internalize fluorescent RIS analogues in in vitro cultures. Rabbit bone marrow cells were treated with FAM-RIS or AF647-RIS for 24 hours, and intracellular uptake of fluorescent RIS analogues was detected by flow cytometry (5*A, B*). AF647-RIS was more readily detectable than FAM-RIS, with relative fluorescence units approximately 20-fold higher for AF647-RIS than for FAM-RIS at all concentrations. Immunofluorescence staining for CD14 revealed that both FAM-RIS and AF647-RIS were predominantly internalized by CD14^high^ monocytes. At all concentrations, the average amount of FAM-RIS or AF647-RIS internalized was higher in CD14^high^ cells than in CD14^neg/low^ cells. Treatment with 10 nM, 100 nM, or 1 µM AF647-RIS resulted in an increase in mean fluorescence intensity over vehicle control of 16.8 ± 4.7-fold for CD14^high^ versus 1.4 ± 0.0-fold for CD14^neg/low^, 143.6 ± 38.9-fold for CD14^high^ (*p* < .01) versus 6.0 ± 1.0-fold for CD14^neg/low^, and 601.3 ± 138.4-fold for CD14^high^ (*p* < .001) versus 26.0 ± 3.0-fold for CD14^neg/low^, respectively (mean ± SD, *n* = 3). Confocal microscopy analysis (see 5*C*) confirmed intracellular uptake of AF647-RIS preferentially by CD14^+^ rabbit bone marrow cells. To determine whether similar effects are seen with human bone marrow cultures in vitro, bone marrow cells obtained from a patient undergoing total hip replacement surgery were treated in vitro with fluorescent RIS analogues for 24 hours, and cellular uptake was determined by flow cytometry. Human bone marrow cells demonstrated similar or even somewhat higher levels of BP uptake compared with rabbit cells (see 5*D*), with 10 nM, 100 nM, or 1 µM AF647-RIS resulting in an increase in mean fluorescence intensity over vehicle control of 65.4 ± 8.4-fold for CD14^high^ versus 2.0 ± 0.1-fold for CD14^neg/low^, 399.7 ± 163.8-fold for CD14^high^ (*p* < .001) versus 9.1 ± 1.6-fold for CD14^neg/low^, and 686.9 ± 69.8-fold for CD14^high^ (*p* < .001) versus 33.3 ± 0.8-fold for CD14^neg/low^, respectively (mean ± SD, three replicates). Finally, rabbit bone marrow cells treated with FITC-dextran, a marker of fluid-phase endocytosis, showed preferential uptake of FITC-dextran by CD14^high^ monocytes (see 5*D*), similar to the uptake of fluorescently labeled RIS.

**Fig. 5:**
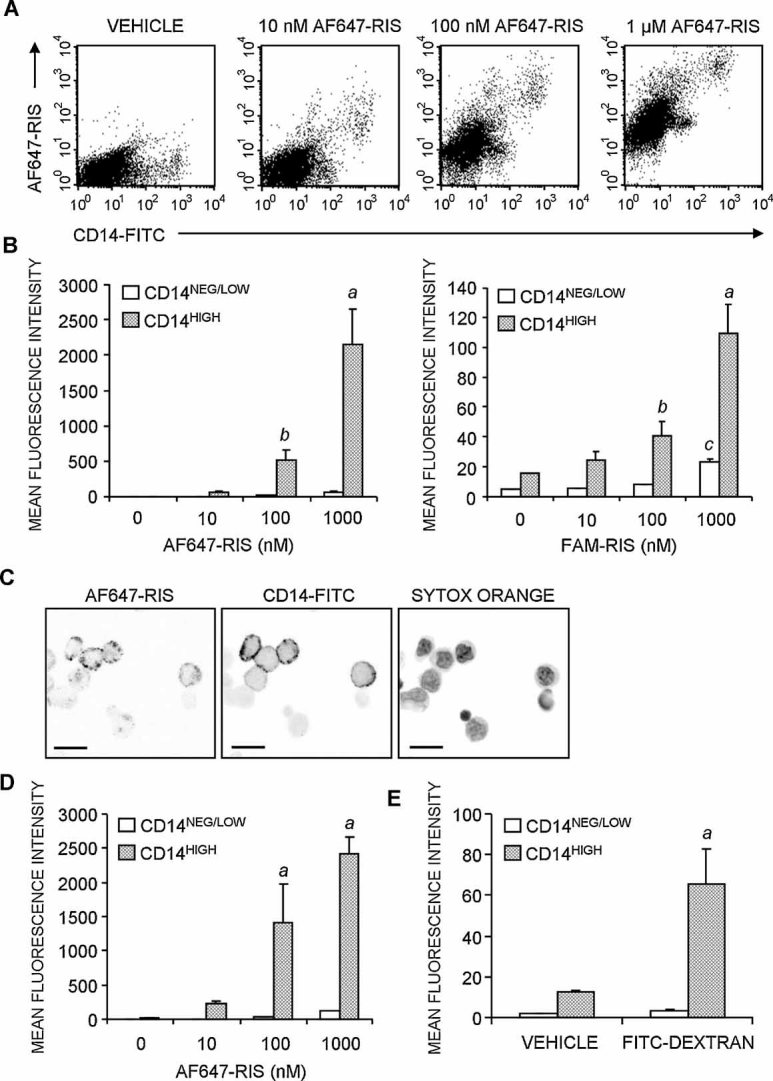
FAM-RIS and AF647-RIS uptake by rabbit bone marrow cells in vitro. Bone marrow cells isolated from the long bones of newborn rabbits by scraping out the marrow (*A–C*, *E*) or isolated from a bone marrow aspirate of a patient undergoing total hip replacement surgery (*D*) were treated in vitro with vehicle only; with 10 nM, 100 nM, or 1 µM AF647-RIS (*A–D*) or FAM-RIS (*A*, *B*); or with 10 µg/mL FITC-dextran (*E*) for 24 hours. Cells then were stained with anti-CD14-APC for FAM-RIS- and FITC-dextran-treated cells or with anti-CD14-FITC for AF647-RIS-treated cells. (*A*) Representative flow cytometry profiles showing AF647-RIS uptake. (*B*) Quantification of flow cytometry results of rabbit bone marrow cells, shown as mean fluorescence intensity expressed as mean ± SD (*n* = 3). (*C*) Confocal microscopic image (1 µm optical section) showing intracellular uptake of AF647-RIS by CD14^+^ cells. Bar = 10 µm. (*D*) Quantification of AF647-RIS uptake by human bone marrow cells by flow cytometry. Results are shown as mean fluorescence intensity and expressed as mean ± SD (three replicates). (*E*) Quantification of flow cytometry analysis of FITC-dextran uptake, shown as mean fluorescence intensity and expressed as mean ± SD (*n* = 3). *a:* *p* < .001; *b:* *p* < .01 versus control and versus respective CD14^neg/low^ cells; *c:* *p* < .05 versus control.

### Uptake of fluorescent RIS analogues by bone marrow cells in vivo

**Fig. 6:**
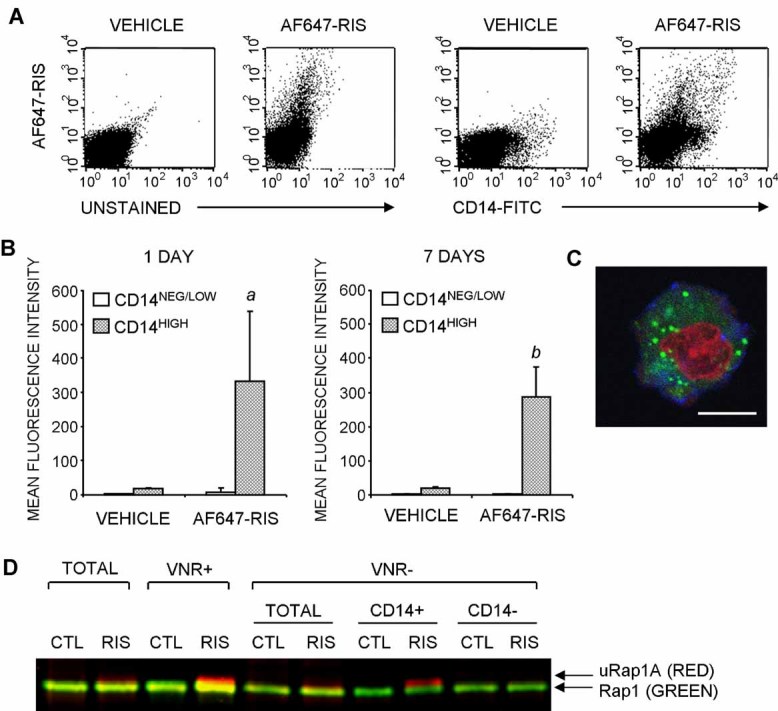
AF647-RIS and RIS uptake by rabbit bone marrow cells in vivo. Newborn rabbits were subcutaneously injected with 0.9 mg/kg AF647-RIS (*A–C*) or 5 mg/kg RIS (*D*) or vehicle and sacrificed 24 houre (*A–D*) or 7 days later (*B*). Bone marrow cells then were isolated from the long bones by scraping out the marrow. (*A–C*) Cells were stained with anti-CD14-FITC and analyzed by flow cytometry and confocal microscopy. (*A*) Representative flow cytometry profiles showing detectable uptake of AF647-RIS by some bone marrow cells in vivo, a subset of which is CD14^high^. (*B*) Quantification of flow cytometry results of bone marrow cells analyzed 24 hours (*left*) and 7 days (*right*) after administration of AF647-RIS. Results are shown as mean fluorescence intensity and expressed as mean ± SD (*n* = 3). *a:* *p* ≤ .01; *b:* *p* < 0.001 versus control and versus CD14^neg/low^ cells. (*C*) 1 µm optical section showing AF647-RIS uptake by a CD14^+^ cell analyzed following flow cytometric sorting of CD14^high^ AF647-RIS^+^ cells (*green:* AF647-RIS; *blue:* CD14-FITC; *red:* sytox orange; bar = 10 µm). (*D*) Osteoclasts were isolated using anti-VNR magnetic bead separation, followed by isolation of monocytes from the VNR-negative fraction by anti-CD14 magnetic beads. Cells were lysed, and unprenylated Rap1A and total Rap1 were detected by SDS-PAGE and Western blotting. *Red:* Unprenylated Rap1A (uRap1A); *green:* Rap1.

To investigate whether any bone marrow cells other than osteoclasts showed detectable levels of uptake of fluorescent RIS, newborn rabbits received a single injection with 0.9 mg/kg AF647-RIS (molar equivalent to 0.2 mg/kg RIS) and after 24 hours or 7 days, bone marrow cells were analyzed for AF647-RIS uptake. AF647-RIS was used because this compound showed far greater sensitivity for detecting intracellular uptake by rabbit bone marrow cells in vitro than FAM-RIS (see Fig. 5B). Uptake in vivo was detected in a subset of bone marrow cells by flow cytometry (6*A, B*). Immunostaining for CD14 revealed that the major cell type that had internalized AF647-RIS were CD14^high^ monocytes, similar to when bone marrow cells were treated with AF647-RIS or FAM-RIS in vitro. The vast majority of CD14^high^ cells showed detectable uptake of AF647-RIS in vivo. The increase in mean fluorescence intensity over vehicle control of CD14^high^ cells after 24 hours was 18.5 ± 11.3-fold (*p* < .05), which was significantly more (*p* < .05) than the CD14^neg/low^ cells (increase in mean fluorescence intensity over vehicle control: 2.5 ± 0.6-fold). AF647-RIS uptake by monocytes was still detectable at similar levels 7 days after administration (see Fig. 5*B*), with an increase in mean fluorescence intensity over vehicle control of 14.3 ± 4.3-fold (*p* = .001) for CD14^high^ cells and 1.7 ± 0.1-fold for CD14^neg/low^ cells. Intracellular uptake of AF647-RIS by CD14^high^ mononuclear cells in vivo was confirmed by fluorescence-activated cell sorting of AF647-RIS^+^CD14^high^ cells followed by confocal microscopic analysis, demonstrating the presence of AF647-RIS within CD14-labeled mononuclear cells (see Fig. 6*C*).

### Inhibition of Rap1A prenylation in rabbit bone marrow cells in vivo in response to RIS

To determine whether efficient uptake of fluorescently labeled RIS by bone marrow monocytes in vivo correlates with an accumulation of unprenylated Rap1A in this cell type in response to RIS, newborn rabbits were treated with RIS or vehicle only, and bone marrow cells were isolated 24 hours later. Osteoclasts were isolated first from the bone marrow cells based on VNR expression, followed by separation of the osteoclast-depleted bone marrow cells into monocytes and non-monocytes based on CD14 expression. At a dose of 5 mg/kg RIS, unprenylated Rap1A was clearly detectable in the osteoclast and monocyte fractions by Western blotting, whereas no unprenylated Rap1A was detected in the non-monocyte fraction or in cells isolated from rabbits treated with vehicle only (see 6*D*).

## Discussion

We report here the use of novel fluorescent RIS analogues to study both the localization and cellular uptake of BP in vivo. We have used RIS conjugated to two different fluorophores, FAM and AF647, employing a newly developed method that allows for stable conjugation to RIS.(19) Labeling with FAM offers the advantage that it emits in the visual spectrum, greatly facilitating analysis of histologic sections and microscopic analysis of intracellular uptake. Moreover, FAM-RIS retains the ability to inhibit protein prenylation both in vitro(19) and in vivo in osteoclasts (this study), suggesting that it closely mimics the pharmacologic characteristics of RIS in vivo. On the other hand, near-infrared imaging using AF647-RIS offers the advantages of high tissue penetration together with minimal tissue autofluorescence, enabling deeper imaging into bones embedded in methyl methacrylate and providing a higher sensitivity of detection than FAM-RIS. The latter property is crucial for permitting the study of cellular uptake of AF647-RIS in a clinically relevant dose range. AF647-RIS also has the advantage over the near-infrared pamidronate analogues Osteosense680 and Osteosense750(17,18) of being readily detectable on standard flow cytometers and confocal microscopes equipped with a 633 nm laser.

We showed previously, by measurement of the retention time on hydroxyapatite columns, that the affinity of FAM-RIS for bone mineral in vitro is comparable, albeit somewhat lower, to that of unlabeled RIS.(19) In this study, these findings have been confirmed using an in vitro HAP crystal growth assay as a model to determine the relative affinity for bone mineral,(20) which demonstrated that both FAM-RIS and AF647-RIS significantly inhibited HAP crystal growth. Furthermore, AF647-RIS was retained in skeletal tissue up to at least 1 week following administration. AF647-RIS also was detectable in kidneys 1 day after administration, consistent with urinary excretion of BP that is not taken up by skeletal tissue following administration.(26) After 1 week, AF647-RIS was no longer detectable in the kidneys, and the liver and spleen did not show significant retention of AF647-RIS at either time point. These findings show that the tissue distribution of AF647-RIS is characteristic of BP drugs,(1,26) suggesting that the pharmacokinetic properties are predominantly conferred by the two phosphonate groups, with little influence of the conjugated side chain.

Histologic analysis revealed that both fluorescent analogues of RIS bound to bone surfaces. The absence of FAM-RIS in nonmineralized cartilage of the growth plate, together with the loss of FAM-RIS labeling of bone surfaces following decalcification and the lack of retention of unconjugated fluorescein in bone, confirmed that FAM-RIS was bound to bone mineral in vivo through the BP moiety of the molecule. More detailed analysis of the distribution of fluorescent RIS analogues on bone surfaces revealed its presence in osteocytic lacunae. In particular, fluorescently labeled RIS was present in lacunar walls close to the bone surface, surrounding osteocytes that appeared to be in the process of becoming entombed, and in lacunae close to vascular channels within cortical bone. Moreover, a clear inverse relationship was found between the distance of the lacuna to the nearest bone or vascular channel surface and the extent of AF647-RIS labeling in terms of both the percentage of positively labeled osteocytic lacunae at a given distance and the mean intensity of labeling. No uptake of BP by osteocytes was observed, although we have found that confocal imaging of these specimens is a relatively insensitive method for assessing intracellular fluorescence (compared with flow cytometry, for example), so we cannot exclude the possibility of intracellular uptake. However, irrespective of cellular uptake, the presence of BP lining the walls of osteocytic lacunae adds credence to previous studies demonstrating prosurvival effects of BPs on osteocytes in vivo mediated through an extracellular mechanism.(27,28) It has been proposed that BPs stimulate the opening of connexin-43 hemichannels and intracellular calcium influx, followed by the rapid activation of extracellular signal-regulated kinases.(29,30) These previous findings raised the possibility that potential interactions of BPs with osteocytes in vivo may contribute to antifracture efficacy, independent of the classic antiresorptive actions via osteoclasts.(1) Interestingly, our findings that fluorescent RIS analogues label predominantly osteocyte lacunae that are close to bone surfaces exposed to the circulation (i.e., the marrow cavity or vascular channels) indicate that only a limited proportion of osteocytes may be exposed to substantial amounts of BP in vivo. It is possible, though, that with repeated dosing and increasing time, an increasing proportion of the osteocytic network may become coated with BP. Furthermore, since the antiapoptotic effects of N-BPs occur at low (nanomolar) concentrations,(27,29) it is possible that the number of osteocytes that are exposed to such concentrations of BP in vivo may be higher than we can detect by fluorescence microscopy. Nevertheless, our findings that the fluorescent RIS analogues were not evenly distributed throughout the canalicular network raises the possibility that the distribution of different BPs within this canalicular network may vary and may be influenced by their affinity for bone mineral. This remains to be further investigated.

Analysis of intracellular uptake of fluorescent RIS analogues demonstrated that CD14^high^ monocytes internalized relatively large amounts of BP both in vitro and in vivo compared with other bone marrow cell populations. This is consistent with our previous findings that BPs, owing to their negatively charged phosphonate groups, largely depend on fluid-phase endocytosis for their cellular uptake(10) and that highly endocytic cell types such as macrophages and monocytes demonstrate much greater levels of intracellular uptake and are more sensitive to BP than less endocytic cell types such as lymphocytes, osteoblasts, and breast cancer cells in vitro.(2,12) Consistent with this, CD14^high^ bone marrow monocytes showed the highest levels of uptake of FITC-dextran, a marker of fluid-phase endocytosis. Preferential uptake of fluorescently labeled RIS by bone marrow monocytes also correlated with selective accumulation of unprenylated Rap1A (a surrogate marker for inhibition of FPP synthase) in monocytes, as well as osteoclasts, in response to treatment with RIS in vivo, demonstrating the potential relevance of selective uptake of BP by monocytes in vivo. Intracellular levels of AF647-RIS in the monocyte population were maintained for at least 7 days after administration. This suggests that BP administration may have prolonged effects on the population of monocytes in the bone marrow. This finding also raises the possibility that the uptake of BP by monocytes in the bone marrow may be a continual process, perhaps secondary to the release of BP from the bone surface during the resorptive process, as was demonstrated previously in in vitro studies.(2) However, it is at present unclear whether the AF647-RIS-labeled monocytes identified at 7 days after administration are a separate population from those present after 24 hours. Thus the relevance of these in vitro findings needs to be further investigated in vivo. Interestingly, an additional minor subset of bone marrow cells that did not express high levels of CD14 also showed detectable uptake of AF647-RIS in vivo, although the degree of uptake was somewhat lower than in CD14^high^ monocytes. While the identity of these cells remains to be determined, a minor subset of CD14^neg/low^ bone marrow cells also showed more than average uptake following treatment of bone marrow cells with AF647-RIS in vitro in the absence of a mineralized surface, suggesting that this population may comprise other highly endocytic cell types present in the bone marrow, such as immature monocytes not (yet) expressing high levels of CD14.

Taken together, these results demonstrate for the first time that non-osteoclast cells are capable of internalizing BP in vivo. Furthermore, the striking correlation between uptake in vitro and uptake in vivo suggests that uptake in vivo is predominantly determined by the endocytic activity of cells rather than by their localization within bone, although spatial distribution in vivo may to some extent influence the amount of BP uptake of individual cells. In particular, cells in close proximity to resorbing osteoclasts may be exposed to BP released from the bone surface during the resorption process that becomes available for intracellular uptake. In support of this, we recently showed in in vitro studies that J774 macrophages show limited BP uptake from the surface of dentine, but this is increased in the presence of resorbing osteoclasts.(2) This could be a contributing factor in determining in vivo cellular uptake of BP by monocytes.

Sato and colleagues calculated from in vitro bone resorption assays that the concentration of ALN achieved in the resorption lacunae of osteoclasts would be 0.1 to 1 mM if 50% of the mineral-bound ALN is released during the resorption process.(7) However, no data currently exist on in vivo concentrations of BP within the bone marrow. The results presented here show that following a single subcutaneous injection with 0.9 mg/kg AF647-RIS (molar equivalent to ∼0.2 mg/kg RIS) in newborn rabbits, cellular uptake of BP by bone marrow cells, as measured by flow cytometry, is comparable with the extent of cellular uptake by bone marrow cells treated with 10 to 100 nM AF647-RIS for 24 hours in vitro (in the absence of a mineral surface). It is therefore tempting to suggest, based on these findings, that the concentration of BP achieved in the bone marrow microenvironment following a single dose with 0.2 mg/kg is somewhere in the range of 10 to 100 nM over a 24 hour period. RIS and ALN are typically administered orally at a weekly dose of 35 or 70 mg, whereas cancer patients generally receive infusions of 4 mg zoledronic acid once every 3 months or up to 90 mg pamidronate every 3 to 4 weeks. While it is difficult to draw direct comparisons between administration to animals and the clinical dose in humans, especially with regard to the orally administered BPs, a dose of 0.2 mg/kg BP in mice appears to be within the clinically relevant range.

The preferential uptake of BP by monocytes, both in vitro and in vivo, may contribute to or help explain some of the actions of BPs. Most notably, it has become clear in recent years that direct effects of BPs on circulating monocytes appear to be responsible for the activation of peripheral blood Vγ9Vδ2 T cells by N-BPs, which can lead to the development of an acute-phase response.(12–16) This clearly indicates that in patients on intravenous BP therapy, BPs reach a sufficiently high concentration in the circulation to directly affect these cells. Additionally, direct effects of BPs on monocytes/macrophages also may be responsible for (some of) the antitumor activity of these drugs. One possible mechanism in humans is through activation of Vγ9Vδ2 T cells, which possess potent antitumoral activity.(31) In addition, some of the antitumor effects of BPs in both humans and animals may be explained by effects on tumor-associated macrophages (TAMs). TAMs can have various protumoral functions, including promotion of angiogenesis, matrix remodeling (facilitating the invasive process), and suppression of adaptive antitumor immunity.(32) Previous studies showed a dramatic decrease in the number of TAMs in various murine models of cancer following treatment with N-BPs, and this was associated with a decrease in tumor vasculature.(33–35) Finally, previous studies have suggested that BPs may act not only by inhibiting the activity of mature osteoclasts but also by decreasing osteoclastogenesis.(36–38) Our findings suggest that effects on osteoclast formation might be explained by BP uptake into monocytic precursors of osteoclasts in the bone marrow.

In conclusion, fluorescent RIS analogues were found to bind to bone surfaces with minimal retention in nonmineralized tissues (kidneys, liver, spleen) and showed uptake by osteoclasts in vivo. The fluorescent RIS analogues labeled osteocytic lacunae either close to the bone surface or close to vascular channels within cortical bone. Since BPs have been shown to promote osteocyte survival through an extracellular effect,(27–30) it is possible that BP present in the osteocyte lacunae is sufficient to directly maintain the survival of these cells in vivo. However, further studies would be required to confirm this. Furthermore, in addition to intracellular uptake by osteoclasts, bone marrow monocytes internalized relatively large amounts of fluorescently labeled RIS in vivo, suggesting that monocytes also may be directly affected by BPs.
